# Colonization with Enterobacteriaceae producing ESBLs in children attending pre-school childcare facilities in the Lao People's Democratic Republic

**DOI:** 10.1093/jac/dkv021

**Published:** 2015-02-12

**Authors:** Nicole Stoesser, Sivilay Xayaheuang, Manivanh Vongsouvath, Koukeo Phommasone, Ivo Elliott, Carlos del Ojo Elias, Derrick W. Crook, Paul N. Newton, Yves Buisson, Sue J. Lee, David A. B. Dance

**Affiliations:** 1 Nuffield Department of Clinical Medicine and the National Institute for Health Research Oxford Biomedical Research Centre (NIHR-OxBRC), University of Oxford, Oxford, UK; 2 Institut de la Francophonie pour la Médecine Tropicale (IFMT), Vientiane, Lao People's Democratic Republic; 3 Lao-Oxford-Mahosot Hospital-Wellcome Trust Research Unit, Microbiology Laboratory, Mahosot Hospital, Vientiane, Lao People's Democratic Republic; 4 Centre for Tropical Medicine and Global Health, Nuffield Department of Medicine, University of Oxford, Oxford, UK; 5 Mahidol-Oxford Tropical Medicine Research Unit (MORU), Faculty of Tropical Medicine, Mahidol University, Bangkok, Thailand

**Keywords:** resistance, carriage, ESBLE

## Abstract

**Objectives:**

Intestinal carriage constitutes an important reservoir of antimicrobial-resistant bacteria, with some of the highest rates reported from Asia. Antibiotic resistance has been little studied in Laos, where some antibiotics are available without restriction, but others such as carbapenems are not available.

**Patients and methods:**

We collected stools from 397 healthy children in 12 randomly selected pre-school childcare facilities in and around Vientiane. Colonization with ESBL-producing Enterobacteriaceae (ESBLE) and carbapenemase-producing Enterobacteriaceae (CPE) was detected using a disc diffusion screening test and ESBLE were characterized using WGS. Risk factor data were collected by questionnaire.

**Results:**

Ninety-two children (23%) were colonized with ESBLE, mainly *Escherichia coli* carrying *bla*_CTX-M_ and *Klebsiella pneumoniae* carrying *bla*_SHV_ or *bla*_CTX-M_, which were frequently resistant to multiple antibiotic classes. Although residence in Vientiane Capital, foreign travel, higher maternal level of education, antibiotic use in the preceding 3 months and attending a childcare facility with a ‘good’ level of hygiene were all associated with ESBLE colonization on univariable analysis, a significant association remained only for antibiotic use when a stepwise approach was used with a multivariate random-effects model. WGS analysis suggested transmission in both childcare facilities and community settings.

**Conclusions:**

The high prevalence of paediatric colonization with ESBLE in Laos, one of the highest reported in Asia, is probably the result of inappropriate antibiotic use. Paediatric colonization with CPE was not identified in this study, but it is important to continue to monitor the spread of antibiotic-resistant Enterobacteriaceae in Laos.

## Introduction

ESBL-producing Enterobacteriaceae (ESBLE) are increasingly common, resulting in the wider clinical use of carbapenems. High intestinal ESBLE colonization prevalence rates have been reported in Asia, with CTX-M enzymes predominating.^1^ Despite limited regulation of antimicrobial prescribing in Laos, the emergence of ESBLE appears to have lagged behind other countries in the region.^2^ Young children represent 11% of the population in Laos^3^ and may act as a reservoir for antimicrobial-resistant organisms.^4^

The aims of this study were to: (i) determine the intestinal colonization prevalence of ESBLE and carbapenemase-producing Enterobacteriaceae (CPE) in Lao children; (ii) investigate risk factors for ESBLE/CPE; and (iii) characterize the genotype and genetic relatedness of any isolates detected.

## Patients and methods

Faecal samples were collected from children ≤6 years of age in six pre-school childcare facilities in Vientiane Capital (VTE) and six in Vientiane Province (VTP), central Laos, in March–June 2011. The childcare facilities, each attended by >50 children, were selected using a random number generator.

Epidemiological data for putative risk factors were assessed by questionnaire (see Supplementary section S1 for a list of variables/translated version of questionnaire; available as Supplementary data at *JAC* Online). A hygiene category (good/adequate/poor) was assigned to each childcare facility (WHO guidance).^5^

Faecal samples were diluted 1 : 10 in saline and incubated on MacConkey agar with 10 μg cefpodoxime and imipenem discs (Oxoid, Basingstoke, UK) within 24 h of collection. Any Gram-negative/oxidase-negative bacilli growing within the antibiotic zones of inhibition and representing distinct colonial morphotypes were tested for ESBL production.^6^ Species identification for ESBLE was performed with the API20E system (bioMérieux, Marcy-l'Étoile, France). Isolates growing within the imipenem inhibition zone were screened using a modified Hodge test^6^ with 10 μg imipenem and meropenem discs. Additional disc diffusion testing was undertaken for amoxicillin/clavulanate, cefotaxime, ceftriaxone, ceftazidime, co-trimoxazole, chloramphenicol, ofloxacin, gentamicin and nitrofurantoin.^6^

DNA was extracted from ESBLE isolates using Quickgene (Fujifilm, Japan) with an additional mechanical lysis step (FastPrep, MP Biomedicals, USA) and sequenced on the Illumina HiSeq 2000, generating 100 base paired-end reads. Sequencing data are available at the European Nucleotide Archive (http://www.ebi.ac.uk/ena/data/view/PRJEB3980).

Reference-based mapping was used to identify nucleotide-level variation [single nucleotide variants (SNVs)] for *Escherichia coli* and *Klebsiella pneumoniae* isolates (Supplementary section S2). Maximum-likelihood phylogenies for each species were created using PhyML.^7^

Reads were *de novo* assembled into contigs using optimized parameters with Velvet/VelvetOptimiser.^8,9^ BLASTn was used to identify the presence of resistance gene variants in the contigs for each isolate (see Table S1 for a list of variants).^10^ Overlapping, partial matches across several contigs representing >80% sequence homology were aligned and this alignment was re-blasted against the database to determine which variant was present.

MLST was similarly determined for *E. coli* and *K. pneumoniae* (Achtman/Pasteur schemes); a match to all reference loci was taken as confirmation of species identification.^11,12^ For species identification of non-*E. coli*/*K. pneumoniae* isolates, sequences for 16S rDNA (using *rrsA* as the reference) were extracted using BLASTn, with top hits analysed using the SeqMatch function at the Ribosomal Database Project (http://rdp.cme.msu.edu/seqmatch/seqmatch_intro.jsp).^13^

Demographic/risk factor data were summarized as frequency (%) or median (IQR). Exact binomial CIs (95%) were calculated for prevalences. Groups were compared using the *χ*^2^ test, Fisher's exact test or the Mann–Whitney *U*-test. Independent risk factors for ESBLE colonization were determined by multivariate analysis using a random-effects model stratified by childcare facility, including all variables with *P* < 0.20 on univariable analysis plus variables previously reported as risk factors for ESBLE colonization (age, history of hospitalization in the previous year and history of urinary tract infection in household). *P* values <0.05 were considered statistically significant and a stepwise approach was used to eliminate non-significant variables from the model.

Statistical calculations were performed with STATA, versions 11 and 13 (StataCorp, College Station, TX, USA).

### Ethical approval

The study was approved by the National Ethics Committee of the Ministry of Health, Lao PDR and by the Oxford Tropical Research Ethics Committee. Consent for enrolment in the study was obtained on behalf of all participants from their parents/guardians.

## Results

In total, 397 individuals were sampled (202 in VTE and 195 in VTP). The median age of participants was 4.2 years (IQR: 3.4–5.2 years) (epidemiological characteristics are summarized in Table S2).

Of 236 colonies growing within cefpodoxime zones, 100 (42.4%) from 92 children were ESBLE (8 children yielded two ESBLE species). Of these, 78 were *E. coli*, 18 *K. pneumoniae*, 3 *Enterobacter* spp. and 1 remained unidentified. No CPE were detected. High levels of concomitant resistance to other antimicrobials were observed (Table 1).

**Table 1.** Antimicrobial susceptibilities of ESBLE isolated

Antimicrobial	*E. coli*, *n* = 78		*K. pneumoniae*, *n* = 18		Other species, *n* = 4		Total, *n* = 100	
	R, *n* (%)	I, *n* (%)	R, *n* (%)	I, *n* (%)	R, *n* (%)	I, *n* (%)	R, *n*	I, *n*
Amoxicillin/clavulanate	6 (8)	35 (45)	0 (0)	0 (0)	3 (75)	1 (25)	9	36
Cefotaxime	76 (97)	1 (1)	11 (61)	7 (39)	4 (100)	0 (0)	91	8
Ceftriaxone	74 (95)	2 (3)	8 (44)	9 (50)	3 (75)	1 (25)	85	12
Ceftazidime	23 (29)	17 (22)	2 (11)	1 (6)	2 (50)	0 (0)	27	18
Co-trimoxazole	58 (74)	0 (0)	18 (100)	0 (0)	4 (100)	0 (0)	80	0
Chloramphenicol	36 (46)	42 (54)	14 (78)	4 (22)	2 (50)	2 (50)	52	48
Ofloxacin	15 (19)	0 (0)	1 (6)	0 (0)	0 (0)	0 (0)	16	0
Gentamicin	33 (42)	1 (1)	4 (22)	0 (0)	3 (75)	0 (0)	40	1
Nitrofurantoin	1 (1)	2 (3)	2 (11)	4 (22)	1 (25)	1 (25)	4	7

R, resistant; I, intermediate.

Overall, the ESBLE faecal colonization prevalence was 23.2% (95% CI 19.1%–27.6%): 29.7% (95% CI 23.5%–36.5%) in VTE and 16.4% (95% CI 11.5%–22.4%) in VTP (*P* = 0.002). Variable colonization prevalence rates were observed between childcare facilities (Figure S1).

On univariable analysis, residence in VTE, foreign travel, antibiotic use in the preceding 3 months, higher maternal level of education and being in a childcare facility with a ‘good’ level of hygiene were all significantly associated with ESBLE colonization (Table S3). A significant association remained only for antibiotic use when a stepwise approach was used with the multivariate random-effects model, increasing the odds 2-fold (OR 2.11, 95% CI 1.21–3.68; *n* = 347 in the final model, stratified by childcare facility).

For the 77 successfully sequenced *E. coli*, we identified 33 different STs, 3 of which were novel. Common STs included: ST38 (11 isolates), ST131 (6), ST10 and ST48 (5 each); only 1 isolate was ST648 (accounting for 19% of clinical isolates in Vientiane previously).^2^ There was no significant difference in the distribution of STs by geographical location (*P* = 0.40). For the 18 *K. pneumoniae*, we identified 11 STs, 4 of which were novel. Only one ST was observed in both settings (ST34).

**Figure 1.**
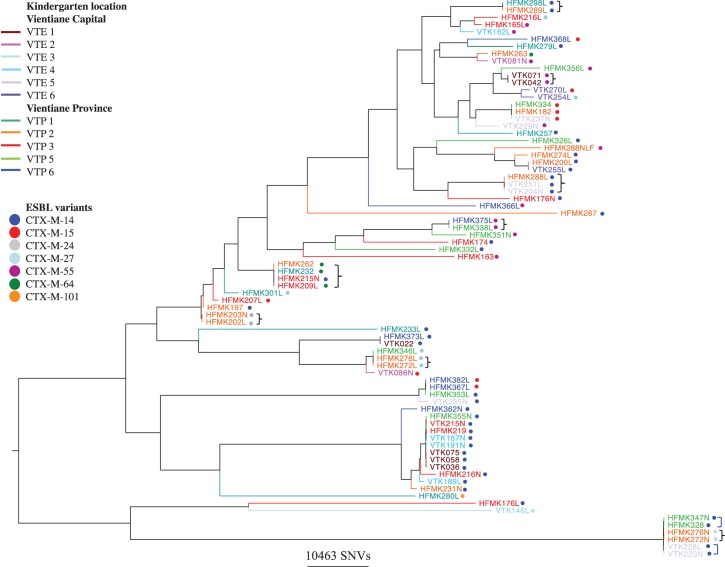
Phylogenetic relationships between ESBL-producing *E. coli* strains isolated from Lao children and associated ESBL gene variants. A curly, black bracket represents clusters of isolates with no SNVs between them and a square, blue bracket represents pairs of isolates with one SNV between them.

Some 141 042 variable sites were identified amongst the 77 mapped *E. coli* strains, with genetically identical clusters (no SNV differences) amongst isolates from children attending the same childcare facility as well as amongst those attending different childcare facilities (Figure 1). Amongst the 18 *Klebsiella* isolates, 269 956 variable sites were identified, with a high degree of relatedness only amongst isolates obtained from children in the same childcare facility (Figure S2).

Amongst ESBL-producing *E. coli*, resistance was universally explained by *bla*_CTX-M_ as follows: *bla*_CTX-M-14_ (36/77 isolates; 47%), *bla*_CTX-M-15_ (10/77; 13%), *bla*_CTX-M-55_ (13/77; 17%), *bla*_CTX-M-27_ (9/77; 12%), *bla*_CTX-M-64_ (5/77; 6%), *bla*_CTX-M-24_ (3/77; 4%) and *bla*_CTX-M-101_ (1/77; 1%). Amongst the *K. pneumoniae* isolates, 13/18 (72%) had *bla*_SHV-2A_ and 5 had *bla*_CTX-M-14_ (28%). One *Enterobacter* sp. isolate contained *bla*_CTX-M-63_ and one *bla*_SHV-2_; in the third isolate, no class A ESBLs were detected.

## Discussion

ESBLE are an emerging cause of infection in Vientiane,^2^ but there are no previous studies of gastrointestinal ESBLE colonization in Laos. In this study, a substantial proportion of healthy children ≤6 years (23%) were ESBLE colonized; colonization prevalence was twice as high in VTE (30%) versus VTP (16%). No CPE were identified, despite their detection in neighbouring countries.^14,15^

ESBL-producing *E. coli* and *K. pneumoniae* were the most common colonizers in our study (78% and 18% of isolates, respectively). CTX-M genes were the most prevalent mechanisms, with a similar distribution of variants in *E. coli* as found previously in local clinical samples,^2^ and some additional ones, including CTX-M-24, 64 and 101. By contrast, ESBL-producing *K. pneumoniae* contained only CTX-M-14 and SHV-2A variants. The genetic relatedness of some *E. coli* and *K. pneumoniae* strains within the same childcare facilities suggests localized transmission. Additionally, for ESBL-producing *E. coli*, genetically identical strains were shared between children attending different childcare facilities and in one instance between children attending childcare facilities in both VTP and VTE, suggesting a wide and fluid colonization reservoir.

Use of antibiotics in the 3 months prior to sampling was identified as the only risk factor that remained significantly associated with ESBLE colonization in the multivariate analysis, almost doubling the odds, consistent with other data.^16,17^ Concomitant antimicrobial resistance was common (Table 1) and may contribute to selection of ESBLE. Antibiotic selection pressures may be driven by a number of factors, including inappropriate self-medication, lack of prescribing regulations, substandard/falsified medicines containing antibiotics and agricultural use.

There are several limitations to our study. The sampling of a small number of childcare facilities may have introduced a ‘cluster effect’, given that ESBLE appear to be transmitted within these. We did, however, sample a highly divergent set of ESBL-producing *E. coli*/*K. pneumoniae* strains, as evidenced by the phylogenies. Childcare is not subsidized and the sampling may have been biased towards wealthier individuals, although 19% of participants came from households earning <65 USD/month. Our ESBLE screening method, selected for its practicability, probably lacks a degree of sensitivity for individuals colonized with small numbers of organisms and we may therefore have underestimated the colonization prevalence and strain diversity in our population. This may also have influenced our risk factor analysis, as preceding antibiotic use may be associated with an increased level of ESBLE colonization rather than ESBLE colonization *per se*. Finally, our WGS mapping-based approach would fail to recognize non-core genome genetic diversity and may inaccurately support a transmission hypothesis. However, similar approaches have been used to reliably assess transmission of hospital-associated pathogens^18^ and *Mycobacterium tuberculosis* in the community.^19^

Although there is some evidence that ESBLE have emerged relatively late in Laos compared with neighbouring countries,^20^ they are now well established in a healthy community reservoir, highlighting the need for ongoing surveillance and regulation of antimicrobial use. Treatment options for ESBLE infections in Laos are limited, given that carbapenems and nitrofurantoin are not currently available.

## Funding

This work was supported by the Wellcome Trust of Great Britain, through funding for the Lao-Oxford-Mahosot Hospital-Wellcome Trust Research Unit. N. S. is a Wellcome Trust/University of Oxford-funded doctoral research fellow. S. X. undertook part of this study as a project in fulfilment of the requirements of the Master in Tropical Medicine and International Health of IFMT. C. d. O. E. is funded by the UKCRC MMM Consortium. D. W. C. is part funded by the NIHR Oxford Biomedical Research Centre and is an NIHR Senior Investigator.

## Transparency declarations

None to declare.

## Supplementary data

Supplementary sections 1 and 2, Tables S1 to S3 and Figures S1 and S2 are available as Supplementary data at *JAC* Online (http://jac.oxfordjournals.org/).

Supplementary Data
