# Supplementary material for: Colonization with Enterobacteriaceae producing ESBLs in children attending pre-school childcare facilities in the Lao People's Democratic Republic
Source: J Antimicrob Chemother. 2015 Feb 12;70(6):1893–7. doi: 10.1093/jac/dkv021 (PMC4498295; doi:10.1093/jac/dkv021)
Supplement: Supplementary Data [file supp_70_6_1893__index.html]

Colonization with Enterobacteriaceae producing ESBLs in children attending pre-school childcare facilities in the Lao People's Democratic Republic — Colonization with Enterobacteriaceae producing ESBLs in children attending pre-school childcare facilities in the Lao People's Democratic Republic — Supplementary Data 

# Colonization with Enterobacteriaceae producing ESBLs in children attending pre-school childcare facilities in the Lao People's Democratic Republic

## Supplementary Data

Supplementary Data

**Files in this Data Supplement:**

- Supplementary Data - Doc file
