# Supplementary material for: Colonization with Enterobacteriaceae producing ESBLs in children attending pre-school childcare facilities in the Lao People's Democratic Republic
Source: J Antimicrob Chemother. 2015 Feb 12;70(6):1893–7. doi: 10.1093/jac/dkv021 (PMC4498295; doi:10.1093/jac/dkv021)
Supplement: Supplementary Data [file supp_dkv021_dkv021supp.doc]

**Supplementary data**

**Supplementary section 1**

The following data were collected by questionnaire for each participant: age; sex; number of children and adults in the household; ethnicity; parents’ occupation and level of education; presence of animals in the household; source of household water supply; socio-economic indicators (income, vehicles, household appliances); travel history; history of hospitalization in the preceding year (prior to the three-month pre-enrolment time period which was an exclusion criterion); history of antibiotics and other medication during the previous three months; history of diarrhoea and fever in the preceding fortnight; history of illness or hospitalization of family members in the previous three months; hygienic practices (hand-washing, toileting).

An English translation of the questionnaire used was as follows:

**No: ...........................**

Date: /__/__/__/ Place: .................................

DEMOGRAPHICS

1. Name: ..............................

2. Date of birth: /__/__/__/

3. Age: ____Years ____Months

4. Sex: ☐Male ☐Female

5. Ethnicity: ☐Lao Loum ☐Lao Soung ☐Lao Theung

Father’s occupation: .............................

Father’s level of education:.....................

Mother’s occupation:..............................

Mother’s level of education:.....................

Telephone number: ....................................

6. Number of people in family: /__/__/ children (less than 15 years)

and: /__/__/ adults

7. Do you keep animals at your house? ☐Yes ☐No

If yes: ☐Dog ☐Cat ☐Birds ☐Others............................

7.1 Do you raise chickens at home? ☐Yes ☐No

8. Source of drinking water: .......................................

9. Family’s socio-economic level

1. What vehicle does the family possess? ☐None ☐Bicycle ☐Motorbike ☐Car

2. Does the family own a refrigerator? : ☐Yes ☐No

3. Monthly family income: ☐< 500,000 kip/month

☐500,000–1,000,000 kip/month

☐1,000,000 –5,000,000 kip/month

☐>5,000,000 kip/month

10. During the last 3 months, has your child travelled outside Laos? ☐Yes ☐No

If yes

10.1. Which country?:....................................

For how long?:............................

Was s/he unwell during the trip? ☐Yes (details)...................... ☐No

10.2. Which country?:....................................

For how long?:............................

Was s/he unwell during the trip? ☐Yes (details)...................... ☐No

PREVIOUS MEDICAL HISTORY OF CHILD:

11. Has the child been admitted to hospital during the past year? : ☐Yes ☐No

☐Don’t know

If yes:

When?: .......................................

Under which speciality?: ................................Which hospital?:......................................

Diagnosis:..................................................

Number of days:..............................

12. Has the child taken any medications in the past 3 months? : ☐Yes ☐No

☐Don’t know

If yes, give details:

Antibiotic ..................

Other ..........................

Date:...........................

13. Has your child had diarrhoea during the past 2 weeks? ☐Yes ☐No ☐Don’t know

If yes, how did you treat it?...............................................

14. Has your child had a fever during the past 2 weeks? ☐Yes ☐No ☐Don’t know

If yes, how did you treat it?...............................................

FAMILY HISTORY:

15. During the past 3 months has anyone in your family had a urinary tract infection?

☐Yes ☐No

16. During the past 3 months has anyone in your family been admitted to hospital?

☐Yes ☐No

If yes:

1. Who?:................................(brother, sister, parent,...)

For what illness? ....................................................

Under which specialty?........................................

Which hospital?:................................

For how long?:...............................................

2. Who?:................................(brother, sister, parent,...)

For what illness? ....................................................

Under which specialty?........................................

Which hospital?:................................

For how long?:...............................................

HYGIENE

17. Does your child often wash his/her hands with soap?: ☐Yes ☐No ☐Sometimes

18. Where do you usually defaecate?

☐Toilet ☐In the forest ☐In fields ☐Other

**Supplementary section 2**

Properly paired sequence reads were mapped using Stampy v1.0.17 (without Burrows-Wheeler Aligner pre-mapping, using an expected substitution rate of 0.01) to species-appropriate reference genomes (*Escherichia coli* CFT073 [GenBank: AE014075.1] and *Klebsiella pneumoniae* MGH78578 [GenBank: CP000647.1].

Repetitive regions of the reference genomes were identified using BLAST, and masked prior to mapping. Single nucleotide variants (SNVs) were determined across all mapped non-repetitive sites using SAM tools (version 0.1.18) mpileup with the extended base-alignment quality flag, after parameter tuning based on bacterial sequences (options '-E -M0 –Q25 -q30 -m2 -D –S' and otherwise default values). GATK v 1.4.21 was used to create VCF files of annotated variant sites. We only used SNVs that were supported by ≥5 reads, including one in each direction. A consensus of ≥0.90 of high quality bases (Phred scaled quality ≥25) was also required to support a SNV, and calls had to be homozygous under a diploid model. Calls required the proportion of bases of Phred quality ≥25 in the 100 base reads spanning the site of interest to be ≥0.35.

For the 77 successfully sequenced *E. coli* isolates, the mean percentage of the reference genome that was called was 74% (range: 69%-81%); for the 18 *K. pneumoniae* isolates it was 87% (82-90%). Reference mapping and *de novo* assembly for a single *E. coli* isolate were poor (low number of sequencing reads [490086] leading to limited mean coverage depth [6.8x]; only 36303 bases *de novo* assembled) – this isolate was therefore excluded from further analysis. The *de novo* assembly for the unidentified isolate suggested that a mixed sample had been sequenced (10.5 megabases in the assembly) – this was therefore also excluded from analysis. For the *de novo* assemblies for all other isolates, the median number of contigs was 249 (74-1658) with a median n50 of 159391 bases (46606-380436 bases; n50 is the largest contig length such that 50% of assembled bases are represented in contigs of this length or longer).

**Table S1. Extended-spectrum beta-lactamase (Class A) resistance mechanisms included in the study resistance database.**

| **Enzyme family** | **Major sub-groups** | **Variants** | **Molecular class** | **Bush-Jacoby functional classification** |
| --- | --- | --- | --- | --- |
| *bla*_ACI_ |  | ACI-1 | A | 2be |
| *bla*_BES_ |  | BES-1 | A | 2be |
| *bla*_BIC_ |  | BIC-1 | A | 2e |
| *bla*_BIL_ |  | BIL-1 (CMY-2) | A | 2ber |
| *bla*_CEPA_ |  | *bla*_CEPA_, *bla*_CEPA_-14, 29, 44, 49, 85 | A | 2e |
| *bla*_CKO_ |  | CKO-1 | A | 2be |
| *bla*_CIA_ |  | CIA-1-4 | A | 2be |
| *bla*_CME_ |  | CME-1, 2 | A | 2be |
| *bla*_CSP_ |  | CSP-1 | A | 2be |
| *bla*_CTX-M_  (*bla*_UOE_, *bla*_KLUC_, *bla*_KLUG_) | CTX-M-1-like  CTX-M-2-like  CTX-M-8-like  CTX-M-9-like  CTX-M-25-like  Hybrids | CTX-M-1, 3, 10, 11, 12, 15, 22, 23, 28, 29, 30, 32, 33, 34, 36, 37, 42, 52, 53, 54, 55/57, 58, 60, 61, 62, 66, 68, 69, 71, 72, 79, 80, 82, 88, 96, 101, 114, 116, 117, 132, 133  CTX-M-2, 4, 5, 6, 7, 20, 31, 35, 43, 44 (TOHO-1), 56, 59, 74, 75, 92, 95, 97, 124, 131  CTX-M-8, 40, 63  CTX-M-9, 13, 14/18, 16, 17, 19, 21, 24, 27, 38, 45 (TOHO-2, 46, 47, 48, 49, 50, 51, 65, 67, 81, 83, 84, 85, 86, 87, 90, 93, 98, 99, 104, 105, 111, 112, 113, 121, 122, 126  CTX-M-25, 26, 39, 41, 89, 91, 94  CTX-M-64, CTX-M-123, CTX-M-78 | A | 2be |
| *bla*_DES_ |  | DES-1 | A | 2be |
| *bla*_ERP_ |  | ERP-1 | A | 2be |
| *bla*_FONA_ |  | FONA-1-6 | A | 2be |
| *bla*_GES_ |  | GES-1-22 | A | 2e, 2be, 2f |
| *bla*_IBC_ |  | IBC-1 (GES-7), IBC-2 (GES-8) | A | 2be |
| *bla*_LUT_ |  | LUT-1 | A | 2be |
| *bla*_ORN_ |  | ORN-1b | A | 2be |
| *bla*_OXY_ | OXY-1  OXY-2  OXY-3  OXY-4  OXY-5  OXY-6 | 1-7  1-13  1  1  1, 2  1-4 | A | 2be |
| *bla*_PER_ |  | PER-1-6 | A | 2be |
| *bla*_PLA_ |  | PLA-2a | A | 2be |
| *bla*_PME_ |  | PME-1 | A | 2be |
| *bla*_RAHN_ |  | RAHN-1, 2 | A | 2be |
| *bla*_SFO_ |  | SFO-1 | A | 2be |
| *bla*_SHV_ |  | SHV-1-3, 5-8, 11, 12, 14, 18, 24-42, 44-51, 53, 55-57, 59-67, 69-72, 77, 84-86, 89, 92-97, 101-105, 108, 109, 112, 121, 122, 128-135 | A | 2b, 2be, 2br |
| *bla*_SPU_ |  | SPU-1 | A | 2be |
| *bla*_TEM_ |  | TEM-1-13, 15-22, 24-26B, 28-40, 42-44, 47-49, 52-55, 7, 63/64, 67-68, 70-72, 75-91, 93-99, 101, 104-134, 136-139, 141-152, 154-160, 162-164, 166-169, 171, 176-178, 183, 185-195, 197-198, 201 | A | 2b, 2be, 2br, 2ber, |
| *bla*_TER_ |  | TER-1 | A | 2be |
| *bla*_TLA_ |  | TLA-1 | A | 2be |
| *bla*_VEB_ |  | VEB-1-7 | A | 2be |
| *cfxA* |  | *cfxA*, *cfxA*3-5 | A | 2be |
| *hugA* |  |  | A | 2be |

**Table S2. Characteristics of children (n=397) sampled in Vientiane Capital (VTE) and Vientiane Province (VTP). Values are frequency (%) unless otherwise specified.**

|  | **Overall** | **Vientiane Capital (N=202)** | | **Vientiane Province (N=195)** | |
| --- | --- | --- | --- | --- | --- |
|  | **n (%)** | **N** | **n (%)** | **N** | **n (%)** |
| **ESBL carriage*** | 92 (23.17) | 202 | 60 (29.70) | 195 | 32 (16.41) |
| **Median age [IQR], years** | 4.17 [3.42-5.17] | 180 | 4.25 [3.50-5.08] | 195 | 4.17 [3.17-5.25] |
| **Male (%)** | 191 (48.11) | 202 | 101 (50.00) | 195 | 90 (46.15) |
| **Ethnic group** |  |  |  |  |  |
| **Lao Loum** | 340 (95.77) | 160 | 158 (98.75) | 195 | 182 (93.33) |
| **Lao Soung (Hmong)** | 2 (0.56) | 160 | 2 (1.25) | 195 | 0 (0) |
| **Lao Theung (Khmu)** | 13 (3.66) | 160 | 0 (0) | 195 | 13 (6.67) |
| **Father’s highest level of education** |  |  |  |  |  |
| **Primary** | 19 (5.56) | 147 | 3 (2.04) | 195 | 16 (8.21) |
| **Secondary** | 199 (58.19) | 147 | 76 (51.70) | 195 | 123 (63.08) |
| **College** | 124 (36.26) | 147 | 68 (46.26) | 195 | 56 (28.72) |
| **Mother’s highest level of education** |  |  |  |  |  |
| **Primary** | 22 (6.43) | 147 | 3 (2.04) | 195 | 19 (9.74) |
| **Secondary** | 221 (64.62) | 147 | 94 (63.95) | 195 | 127 (65.13) |
| **College** | 99 (28.95) | 147 | 50 (34.01) | 195 | 49 (25.13) |
| **Monthly income (Lao kip)** |  |  |  |  |  |
| **<500000** | 71 (18.98) | 179 | 14 (7.82) | 195 | 57 (29.23) |
| **500,000-999,999** | 151 (40.37) | 179 | 49 (27.37) | 195 | 102 (52.31) |
| **1,000,000-5,000,000** | 152 (40.64) | 179 | 116 (64.80) | 195 | 36 (18.46) |
| **Owns a bicycle** | 72 (19.25) | 179 | 23 (12.85) | 195 | 49 (25.13) |
| **Owns a motorcycle** | 362 (96.79) | 179 | 177 (98.88) | 195 | 185 (94.87) |
| **Owns a car** | 74 (19.79) | 179 | 37 (20.67) | 195 | 37 (18.97) |
| **Owns a television** | 355 (94.67) | 180 | 179 (99.44) | 195 | 176 (90.26) |
| **Owns a refrigerator** | 361 (96.27) | 180 | 178 (98.89) | 195 | 183 (93.85) |
| **Median number of individuals in household [IQR]** | 5 [4-6] | 177 | 4 [4-5] | 195 | 5 [4-6] |
| **Tap water in household** | 287 (73.78) | 194 | 161 (82.99) | 195 | 126 (64.62) |
| **Antibiotic use in the preceding three months** | 145 (41.79) | 171 | 81 (47.37) | 176 | 64 (36.36) |
| **Presence of any pet animal or livestock in household** | 223 (75.85) | 99 | 59 (59.60) | 195 | 164 (84.10) |
| **Presence of dog in household** | 94 (31.97) | 99 | 33 (33.33) | 195 | 61 (31.28) |
| **Presence of cat in household** | 37 (12.59) | 99 | 11 (11.11) | 195 | 26 (13.33) |
| **Presence of poultry in household** | 198 (67.35) | 99 | 44 (44.44) | 195 | 154 (78.97) |
| **Foreign travel in last 3 months** | 12 (3.86) | 116 | 9 (7.76) | 195 | 3 (1.54) |
| **History of UTI in the family in past 3 months** | 3 (0.79) | 187 | 3 (1.60) | 195 | 0 (0) |
| **History of hospitalisation in the preceding year** | 55 (18.71) | 99 | 16 (16.16) | 195 | 39 (20.00) |
| **Child usually uses toilet for defaecation** | 352 (94.37) | 178 | 173 (97.19) | 195 | 179 (91.79) |
| **Child washes hands with soap** | 354 (94.91) | 178 | 175 (98.31) | 195 | 179 (91.79) |
| **Diarrhoea within the past 2 weeks** | 31 (8.31) | 178 | 16 (8.99) | 195 | 15 (7.69) |
| **Fever within the past 2 weeks** | 80 (20.57) | 194 | 31 (15.98) | 195 | 49 (25.13) |
| **Kindergarten hygiene level**  **Poor**  **Adequate**  **Good** | 3 (25.0)  3 (25.0)  6 (50.0) | 6  6  6 | 1 (16.7)  2 (33.3)  3 (50.0) | 6  6  6 | 2 (33.3)  1 (16.7)  3 (50.0) |

N= Total number of children with non-missing data; n=number of observations

* ESBL carriage was significantly higher among children in Vientiane capital when compared with children in Vientiane province, p = 0.002

**Table S3. Univariate analysis of risk factors for carriage of ESBLE amongst 397 study children. All variables with p <0.20 (in bold) were included in the multivariable logistic regression model to identify independent predictors of ESBLE carriage. Fully completed questionnaires were available for 86/202 (42.5%) children in VTE and 176/195 (90%) in VTP.**

| **Risk factor** | **ESBLE carrier**  **n/N (%)*** | **ESBLE negative**  **n/N (%)*** | **p-value** |
| --- | --- | --- | --- |
| **Overall** | 92/397 (23.17) | 305/397 (76.83) | - |
| **Age>50 months** | 40/86 (46.51) | 147/289 (50.87) | 0.478 |
| **Male gender** | 41/92 (44.57) | 150/305 (49.18) | 0.437 |
| **Resident in Vientiane Capital** | **60/92 (65.22)** | **142/305 (46.56)** | **0.002** |
| **Ethnic group** |  |  |  |
| **Lao Loum** | 75/78 (96.15) | 265/277 (95.67) |  |
| **Lao Soung (Hmong)** | 1/78 (1.28) | 1/277 (0.36) |  |
| **Lao Theung (Khmu)** | 2/78 (2.56) | 11/277 (3.97) | 0.493**^a^** |
| **Fathers’ education level** |  |  |  |
| **Primary** | 5/69 (7.25) | 14/273 (5.13) |  |
| **Secondary** | 35/69 (50.72) | 164/273 (60.07) |  |
| **College** | 29/69 (42.03) | 95/273 (34.80) | 0.327**^a^** |
| **Mothers’ education level** |  |  |  |
| **Primary** | **5/69 (7.25)** | **17/273 (6.23)** |  |
| **Secondary** | **35/69 (50.72)** | **186/273 (68.13)** |  |
| **College** | **29/69 (42.03)** | **70/273 (25.64)** | **0.021^a^** |
| **Parental income (Kips)/month** |  |  |  |
| **<500,000** | **15/86 (17.44)** | **56/288 (19.44)** |  |
| **500,000-999,999** | **27/86 (31.40)** | **124/288 (43.06)** |  |
| **1,000,000-5,000,000** | **44/86 (51.16)** | **108/288 (37.50)** | **0.067^a^** |
| **Owns a bicycle** | **22/85 (25.88)** | **50/289 (17.30)** | **0.078** |
| **Owns a motorcycle** | 85/86 (98.84) | 277/288 (96.18) | 0.310 |
| **Owns a car** | **23/85 (27.06)** | **51/289 (17.65)** | **0.056** |
| **Owns a television** | **85/86 (98.84)** | **270/289 (93.43)** | **0.055** |
| **Owns a refrigerator** | 85/86 (98.84) | 276/289 (95.50) | 0.205 |
| **> 5 family members in household** | 25/85 (29.41) | 73/287 (25.44) | 0.465 |
| **Median number of members in household [IQR, n]** | 5 [4-6, 85] | 5 [4-6, 287] | 0.622 |
| **Tap water in household** | 62/84 (73.81) | 225/305 (73.77) | 0.994 |
| **Antibiotic use in the 3 months prior to enrolment** | **45/77 (58.44)** | **100/270 (37.04)** | **0.001** |
| **Presence of a pet animal or livestock in household** | 51/65 (78.46) | 172/229 (75.11) | 0.577 |
| **Presence of dog in household** | 22/65 (33.85) | 72/229 (31.44) | 0.714 |
| **Presence of cat in household** | 8/65 (12.31) | 29/229 (12.66) | 0.939 |
| **Presence of poultry in household** | 43/65 (66.15) | 155/229 (67.69) | 0.816 |
| **Foreign travel in the three months preceding enrolment** | **6/66 (9.09)** | **6/245 (2.45)** | **0.023** |
| **History of UTI in the family in past 3 months** | 1/84 (1.19) | 2/298 (0.67) | 0.526 |
| **History of hospitalisation in the preceding year** | 14/65 (21.54) | 41/229 (17.90) | 0.507 |
| **Child usually uses toilet for defecation** | 81/84 (96.43) | 271/289 (93.77) | 0.432 |
| **Child washes hands with soap** | 81/84 (96.43) | 273/289 (94.46) | 0.584 |
| **Diarrhoea within the past 2 weeks** | 7/84 (8.33) | 24/289 (8.30) | 0.993 |
| **Fever within the past 2 weeks** | **23/84 (27.38)** | **57/305 (18.69)** | **0.081** |
| **Kindergarten hygiene level** |  |  |  |
| **Poor** | **7/92 (7.61)** | **63/305 (20.66)** |  |
| **Adequate** | **26/92 (28.26)** | **90/305 (29.51)** |  |
| **Good** | **59/92 (64.13)** | **152/305 (49.84)** | **0.008^a^** |

N= Total number of children with non-missing data; n=number of observations, * unless otherwise specified

^a^ compared using 2 degrees of freedom

**Figure S1. Extended spectrum beta-lactamase-producing Enterobacteriaceae (ESBLE) prevalence rate by kindergarten location.**

**
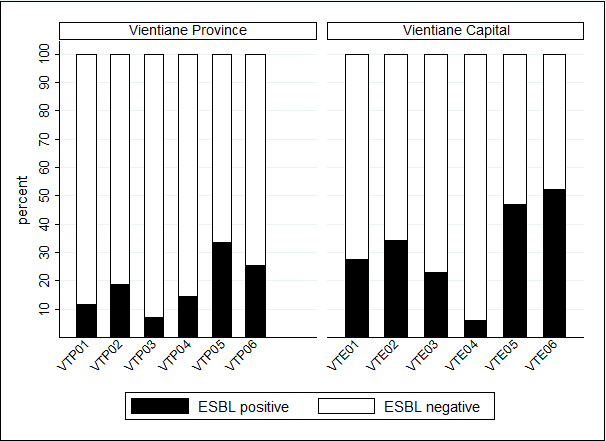
**

**Figure S2. Phylogenetic relationships between extended spectrum beta-lactamase (ESBL)-producing-*Klebsiella pneumoniae* strains isolated from Lao children and associated ESBL gene variants. A curly, black bracket represents clusters of isolates with 0 single nucleotide variants (SNV) between them, and a square, blue bracket represents pairs of isolates with 1 SNV between them.**
